# Rhabdomyolysis Associated with Parainfluenza Virus

**DOI:** 10.1155/2013/650965

**Published:** 2013-06-13

**Authors:** Miltiadis Douvoyiannis, Johanna M. Kielbasa, Gopal M. Chandrasekharan, Cynthia L. Holmes, Michael R. Gomez

**Affiliations:** ^1^Department of Pediatrics, The University of Oklahoma, Tulsa School of Community Medicine, Children's Hospital at Saint Francis, Division of Hospital Pediatrics and Pediatric Infectious Diseases, 4502 East 41st Street, 2A-31, Tulsa, OK 74135, USA; ^2^Department of Clinical and Anatomic Pathology, Children's Hospital at Saint Francis, 2738 East 51st Street, Suite 290, Tulsa, OK 74136, USA

## Abstract

Influenza virus is the most frequently reported viral cause of rhabdomyolysis. A 7-year-old child is presented with rhabdomyolysis associated with parainfluenza type 2 virus. Nine cases of rhabdomyolysis associated with parainfluenza virus have been reported. Complications may include electrolyte disturbances, acute renal failure, and compartment syndrome.

## 1. Introduction

Rhabdomyolysis is characterized by the breakdown of skeletal muscle fibers. It has a diverse etiology including infections, trauma, strenuous exercise, drug reactions, metabolic disorders, and status epilepticus [1]. In contrast with the adults, Mannix et al. in the largest series of pediatric rhabdomyolysis, reported that viral myositis was the most frequent cause, accounting for 38% (73/191) of cases and in particular during the first decade of life [2]. Viruses associated with rhabdomyolysis include influenza types A and B, HIV, enteroviruses, Epstein-Barr, cytomegalovirus, adenovirus, herpes simplex, and varicella virus. Influenza has been considered as the most frequent [1].

## 2. Case Description

A 7-year-old boy was referred by his pediatrician in the autumn, with fever up to 39.2°C for 4 days and lower extremities pain, right knee stiffness, and difficulty in ambulating over the previous 2 days. The child had fatigue, nasal congestion, sore throat, and occasional cough. His sister had rhinorrhea and hoarseness. He had been diagnosed with intermittent asthma and received montelukast daily. No family history of metabolic or neuromuscular diseases was noted. No trauma, increased exercise, insect bites, or urine discoloration was reported. He had no pets and had not traveled recently. His immunizations were up to date except for influenza.

On physical examination he had a temperature of 38.6°C, heart rate of 107 beats/min, respiratory rate of 18/min, blood pressure of 109/70 mm Hg, and oxygen saturation of 100% on room air. The child was unable to walk or stand. Upon palpation, he had severe tenderness over the calves and thighs without any swelling and milder pain in the arms. His abdomen was tender to percussion but soft. He had scattered wheezing. No signs of arthritis or rashes were noted. The remaining of his examination was unremarkable. 

White blood cell count was 3.000/mcl, with 60% neutrophils, 32% lymphocytes, and 7% monocytes. The hemoglobin was 13.5 gr/dL, the hematocrit 41%, and the platelet count 244.000/mcl. C-reactive protein and erythrocyte sedimentation rate were normal. His electrolytes were within normal limits; BUN was 13 mg/dL (normal 5–20), and creatinine was 0.44 mg/dL (normal 0.10–0.80). AST was 623 U/L (normal 0–35); ALT was 105 U/L (normal 0–40). Alkaline phosphatase and total bilirubin were normal. A urinalysis obtained by his pediatrician showed pH 6.5, specific gravity 1.037, 2+ protein, and 3+ hemoglobin. Microscopy showed 2 white blood and 1 red blood cells/high-power field. Myoglobin was detected in the urine. Initial serum creatinine phosphokinase (CPK) was 5,614 U/L (normal <226). 

A nasopharyngeal specimen was tested for various respiratory pathogens by a multiplex nested polymerase-chain reaction assay (FilmArray Respiratory Panels, BioFire Diagnostics Inc., Salt Lake City, UT, USA). It was found nonreactive for influenza A and B, adenovirus, rhinovirus/enterovirus, coronavirus, metapneumovirus, respiratory syncytial virus, Bordetella pertussis, Mycoplasma pneumonia, Chlamydia pneumoniae, and parainfluenza virus types 1, 3, and 4, but it was found positive for parainfluenza type 2. A blood culture drawn on admission remained negative.

Increased hydration without potassium was administered, but no alkalinization of the urine or forced diuresis was required. CPK reached up to 21,425 U/L and AST and ALT up to 843 U/L and 245 U/L, respectively. Potassium peaked at 5.5 mEq/L (normal 3.5–5.0) and phosphorus at 5.1 mEq/L (normal 2.4–4.3). CPK decreased to 6,756, and electrolytes normalized on discharge two days later. The child's clinical condition had much improved. 

## 3. Discussion

There is not a clear distinction in the literature between acute benign myositis and rhabdomyolysis. Some use the term acute benign myositis for uncomplicated cases of muscle inflammation with elevated CPK and arbitrarily reserve the term rhabdomyolysis when, in addition, myoglobinuria is present [12]. Others refer to rhabdomyolysis only when serum CPK is >1000 U/L [2]. Since rhabdomyolysis means disintegration of striated muscle with resultant leakage of muscle cell constituents, we choose to include all reports of clinical myositis with elevated serum CPK.

The first case of rhabdomyolysis associated with parainfluenza was reported in 1976 by McKinlay and Mitchell, in an 8-year-old child who had a benign self-limiting course. Since then, 10 cases have been reported on the PubMed database in the English literature (Table 1). Seven cases including the current one occurred in children, and subsequent discussion refers to them. The median age was 7 years (range 4–10, mean 6.8), and two were girls. All children were healthy except a child with cerebral palsy [3]. All children presented with rhabdomyolysis within 1–5 days after the onset of fever or upper respiratory tract symptoms. Fever was reported in 86% (6/7) [3, 4, 6–8]. Myalgias involved predominantly the calves and thighs in all children but one, for which no specific description of muscle involvement was available [3]. Muscle weakness or difficulty in walking was reported in all the remaining cases and muscle swelling in 33% (2/6).

Similarly, in a review of 39 children with influenza associated myositis/rhabdomyolysis, the median age was 8.5 years (range 2.5–14). The median interval was 3 days after the onset of influenza, fever was present in 74%, calf pain was typically present, and difficulty in walking occurred in 26% and swelling in 13% [12].

None of the children with parainfluenza had dark-colored urine, except for one who presented with the “classic triad” of myalgias, muscle weakness, and dark urine [5]. Two had positive urinalysis for blood [5, present]. These findings are in agreement with a review in which only 3.6% of 191 children with rhabdomyolysis presented with dark urine, and only 1 had the “classic triad.” In addition, more than half of childhood rhabdomyolysis cases may have negative heme dipstick results [2]. 

The median CPK level was 7,563 U/L (range 1,566–50,000, mean 15,508) compared with a median of 4,100 U/L (range 230–1,000,000) in 36 children with influenza [12]. In a series of 18 children with rhabdomyolysis, CPK levels correlated with the development of acute renal failure [13]. In contrast, the need for renal replacement therapy did not correlate with the initial or peak level of CPK in another series of 28 children [14]. In children with parainfluenza the median AST was 204 U/L (range 71–1,040, mean 420). Thrombocytopenia (platelet count 52,000/mcl) was reported in one child [8].

The median time to clinical recovery was 12 days (range 4–52, mean 18.8). In some children complications occurred, but the outcome was favorable. Three out of seven children developed ARF, and two of them received renal replacement therapy [3, 4]. One child died because of cardiorespiratory arrest and brain edema [7]. 

Among patients with rhabdomyolysis associated with influenza, ARF has been reported by Singh et al. in 44% (11/25) of patients and by Agyeman et al. in only 3% (8/311) of children with myositis/rhabdomyolysis [1, 12]. Six out of eight children with influenza ARF required renal replacement therapy [12]. In contrast, it has been reported that none of 73 children with rhabdomyolysis associated with viral myositis, resulted in acute renal failure [2]. In addition, renal replacement therapy was not more frequent in children with rhabdomyolysis precipitated by infection in comparison with other causes [14].

In two cases of parainfluenza precipitating rhabdomyolysis, a dual infection was detected in postmortem specimens. In a child, viral particles consistent with a picornavirus were detected by electron microscopy in the muscles [7]. Chlamydia pneumoniae was detected by PCR in an adult with hemorrhagic pneumonia [11]. Whether coinfection carries a worse prognosis in the context of rhabdomyolysis remains to be clarified. 

The pathogenesis of viral-induced rhabdomyolysis is unclear. Direct viral invasion and toxicity or immunologic mechanisms such as deposition of immune complexes or cross-reactivity have been proposed among others [12]. Recently, it has been suggested that, in genetically susceptible hosts, infection with parainfluenza leads to increased production of interferon-1, a known cause of rhabdomyolysis [4]. Similar histopathologic findings have been reported in both influenza and parainfluenza cases. Diffuse or patchy muscle degeneration and necrosis with little inflammatory infiltration were commonly detected [7, 10, 12]. 

Antiviral treatment such as ribavirin in rhabdomyolysis caused by parainfluenza virus was not reported. The efficacy of antivirals such as neuraminidase inhibitors in rhabdomyolysis caused by influenza is unknown. In addition, recurrent rhabdomyolysis and compartment syndrome, first precipitated by parainfluenza and on a second occasion by influenza, have been described in a child unvaccinated for influenza [15]. Recurrent rhabdomyolysis associated with influenza has been reported in 10 children [12]. Our patient's risk of developing recurrent disease upon subsequent viral infection is unknown. His mother agreed for the child to receive the influenza immunization. Influenza vaccination triggering rhabdomyolysis is an extremely rare event and has occurred in adults on statin therapy [16].

## 4. Conclusion

In conclusion, although mild myalgias are commonly reported and are self-limited in infections caused by parainfluenza virus, worsening pains, difficulty in ambulating, exquisite tenderness to palpation, or muscle swelling should prompt further investigations to detect possible rhabdomyolysis, even in the absence of dark urine. Potential complications include acute renal failure, electrolyte disturbances, and compartment syndrome. Although influenza is the most frequently reported viral cause of rhabdomyolysis, the implementation of laboratory methods such as multiplex polymerase-chain reaction assays may lead to enhanced recognition and characterization of rhabdomyolysis associated with parainfluenza or other pathogens. 

## Figures and Tables

**Table 1:** Characteristics of reported patients with rhabdomyolysis associated with parainfluenza virus.

No./age/sex	Method of detection*/serotype	Muscle involvement	Highest CPK (U/L)	Highest AST (U/L)	Complications	Duration of illness (days)	Outcome	Ref.
1/5 y/M	DFA/1	N/A	22,242	1,040	ARF, respiratory failure	52	Survived	Vrsalovic et al.2007 [3]
2/6 y/F	DFA/1	Calves, thighs, and swelling	50,000	156	ARF, compartment syndrome	37	Survived, recurred with influenza	Ebbeson et al. 2009 [4]
3/10 y/M	DFA/1	Classic triad^‡^	7,563	71	None	14	Survived	Pana et al. 2011 [5]
4/8 y/F	Serology/2	Calves, refusal walking	4,060	204	None	7	Survived	Zvolanek 1984 [6]
5/7 y/M	PCR/2	Calves, thighs, arms, abdomen, and knee stiffness	21,425	843	None	6	Survived	Present
6/4 y/M	Viral culture/3	Calves, thighs knee stiffness, and swelling	1,700	205	ARF, respiratory failure, and brain edema	12	Died	Ueda et al. 1978 [7]
7/8 y/M	Viral culture/4	Calves, thighs	1,566	N/A	None	4	Survived	McKinlay and Mitchell 1976 [8]

8/38 y/M	Serology/2	Diffuse	79,000	1,200	None	10	Survived	O'Connor and Iyer 1982 [9]
9/43 y/M	Serology/3	Legs, trunk, and swelling	105,200	56,800	ARF, hyperkalemia	13	Died	Muto et al. 1987 [10]
10/78 y/M	^§^PCR/4	N/A	706	N/A	Hemorrhagic pneumonia	24	Died	Rubinas et al. 2004 [11]

*Viral culture, DFA, and PCR performed in nasopharyngeal specimens.  ^§^PCR performed in lung tissue.

^‡^Classic triad: myalgias, muscle weakness, and dark urine.

ARF: acute renal failure; CPK: creatine phosphokinase; DFA: direct immune-fluorescence antibody assay; N/A: not available; PCR: polymerase-chain reaction; Ref.: reference; y: years.
